# Mid-ureteric Ectasia Masquerading as Hirschsprung Disease: A Rare Cause of Infantile Large Bowel Obstruction

**DOI:** 10.7759/cureus.104486

**Published:** 2026-03-01

**Authors:** Greeshma Suresh, Sarita Chowdhary, Ajit K Vidhyarthy, Umang K Agrawal, Surajit Das, Pranav K Yadav

**Affiliations:** 1 Pediatric Surgery, Institute of Medical Sciences, Banaras Hindu University, Varanasi, IND; 2 General Surgery, Institute of Medical Sciences, Banaras Hindu University, Varanasi, IND

**Keywords:** congenital anomalies of the kidney and urinary tract (cakut), hirschprung’s disease, infant, megaureter, ureteric cyst, ureteric ectasia

## Abstract

Mid-ureteric dilatation with stenosis is extremely rare in children. We discuss a case of a three-month-old infant presenting with a large bowel obstruction and a left-sided abdominal lump. While the large bowel was grossly normal on exploration, a cystic dilatation in the middle of the ureter was causing colonic compression, with the proximal and distal ureteric segments being normal in diameter and exhibiting normal peristaltic activity. Thus, an unusual segmental cystic dilatation of the ureter with stenosis was noted. Excision of the dilated segment of the ureter and end-to-end ureteric anastomosis was performed. Though rare, mid-ureteric ectasia can masquerade as colonic obstruction and should be considered as a differential diagnosis for constipation and left-sided abdominal masses in infancy.

## Introduction

Megaureter is defined as a ureter with a diameter ≥7 mm. It may be classified as primary or secondary and further categorized as refluxing, obstructed, non-refluxing obstructed, or non-refluxing unobstructed. In most cases, the dilatation involves the entire length of the ureter. However, segmental megaureter, also described as segmental cystic ureteric dilatation, is a rare entity with only a few reported cases in the literature [1,2]. The distal ureter in these patients may be normal, stenotic, or atretic, and some cases have been associated with a duplex collecting system on the affected side [3].

Congenital anomalies of the kidney and urinary tract (CAKUT) account for more than 50% of abdominal masses detected in neonates and occur in approximately 0.5% of all pregnancies [4,5]. Most neonatal urinary tract masses arise from posterior urethral valves, pelviureteric junction obstruction, or megaureter [4]. Infants with CAKUT typically present with urinary symptoms such as anuria, dribbling, hematuria, or a palpable lumbar mass; presentation with bowel obstruction is uncommon [5].

We report a rare case of segmental ureteric dilatation presenting as colonic obstruction.

## Case presentation

A three-month-old male infant presented to the Pediatric Surgery OPD at the Institute of Medical Sciences, Varanasi, India, with progressively increasing abdominal distension for three days and non-passage of stools for five days. There was no history of excessive crying, fever, vomiting, skin rash, or urinary complaints.

Antenatal and birth history

Antenatal ultrasonography performed at six months of gestation revealed a twin pregnancy, with one twin showing abdominal distension. No antenatal intervention was undertaken. The twins were delivered via normal vaginal delivery at 34 weeks of gestation. Both babies cried immediately after birth. The presenting infant weighed 1.4 kg, and the co-twin weighed 1.8 kg. Both neonates were kept under observation for three weeks postnatally. The family reported abdominal distension since birth in the presenting infant. Meconium passage occurred on day 2 of life. Subsequently, the child passed stools after feeds during the first two months and two to three times per day during the third month of life.

Clinical examination

On examination, the infant was alert, afebrile, pink, and adequately hydrated, lying comfortably in the mother’s lap. There was no cyanosis or icterus. Vital signs were stable, with a heart rate of 110 beats per minute and a respiratory rate of 40 breaths per minute. Abdominal examination revealed a soft, non-tender, distended abdomen. A firm oblong mass measuring approximately 4 × 5 cm was palpable in the left iliac fossa, clinically resembling a dilated, loaded sigmoid colon. The rest of the abdomen was tympanic, while the lump was dull on percussion. The perineum appeared normal. Per-rectal examination revealed fecal loading, followed by passage of stools. Systemic examination was otherwise unremarkable. Laboratory investigations were within normal limits.

Radiological evaluation

**Figure 1:**
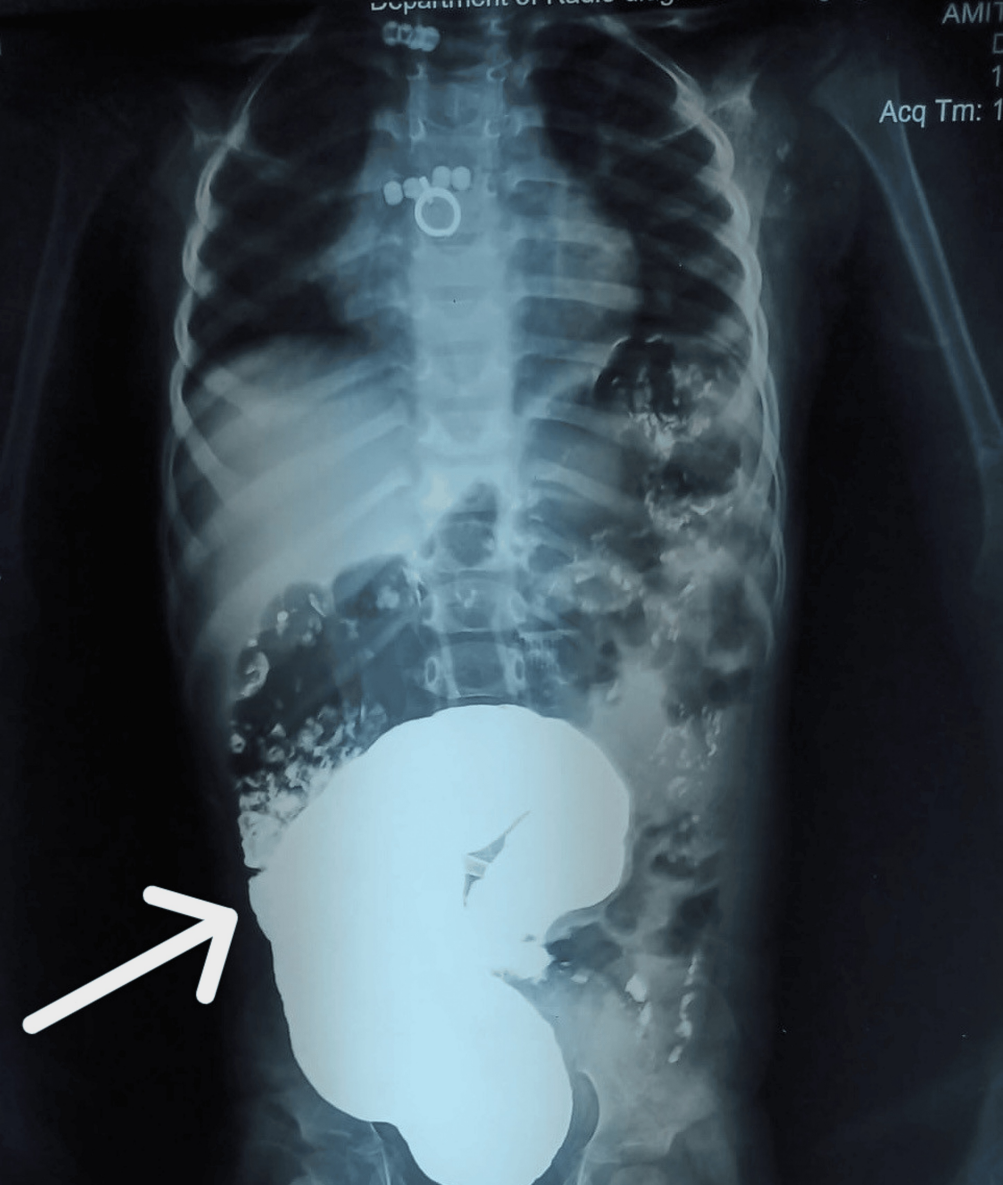
Contrast radiograph demonstrating extrinsic compression of the sigmoid colon Contrast study of the abdomen showing a smooth, well-defined impression over the sigmoid colon (white arrow), produced by an adjacent extrinsic mass effect. The absence of mucosal irregularity or intrinsic narrowing suggests an external compressive etiology rather than primary colonic pathology, consistent with compression by a segmentally dilated ureter.

Ultrasonography of the abdomen showed a dilated colon along with dilatation of the renal pelvis and mid ureter. Contrast enema demonstrated a dilated colon with significant contrast retention and no definite transition zone (Figure 1).

Despite regular enemas, the child continued to have recurrent abdominal distension and decreased stool frequency. A provisional diagnosis of Hirschsprung disease or congenital band obstruction was made, and exploratory laparotomy with possible colonic biopsy and colostomy was planned.

Operative findings

Intraoperatively, the bowel appeared grossly normal, with mild proximal colonic dilatation. A transversely oriented cystic mass measuring approximately 4 × 7 cm was identified, compressing the distal sigmoid colon. A well-defined plane was present between the mass and the colon.

**Figure 2:**
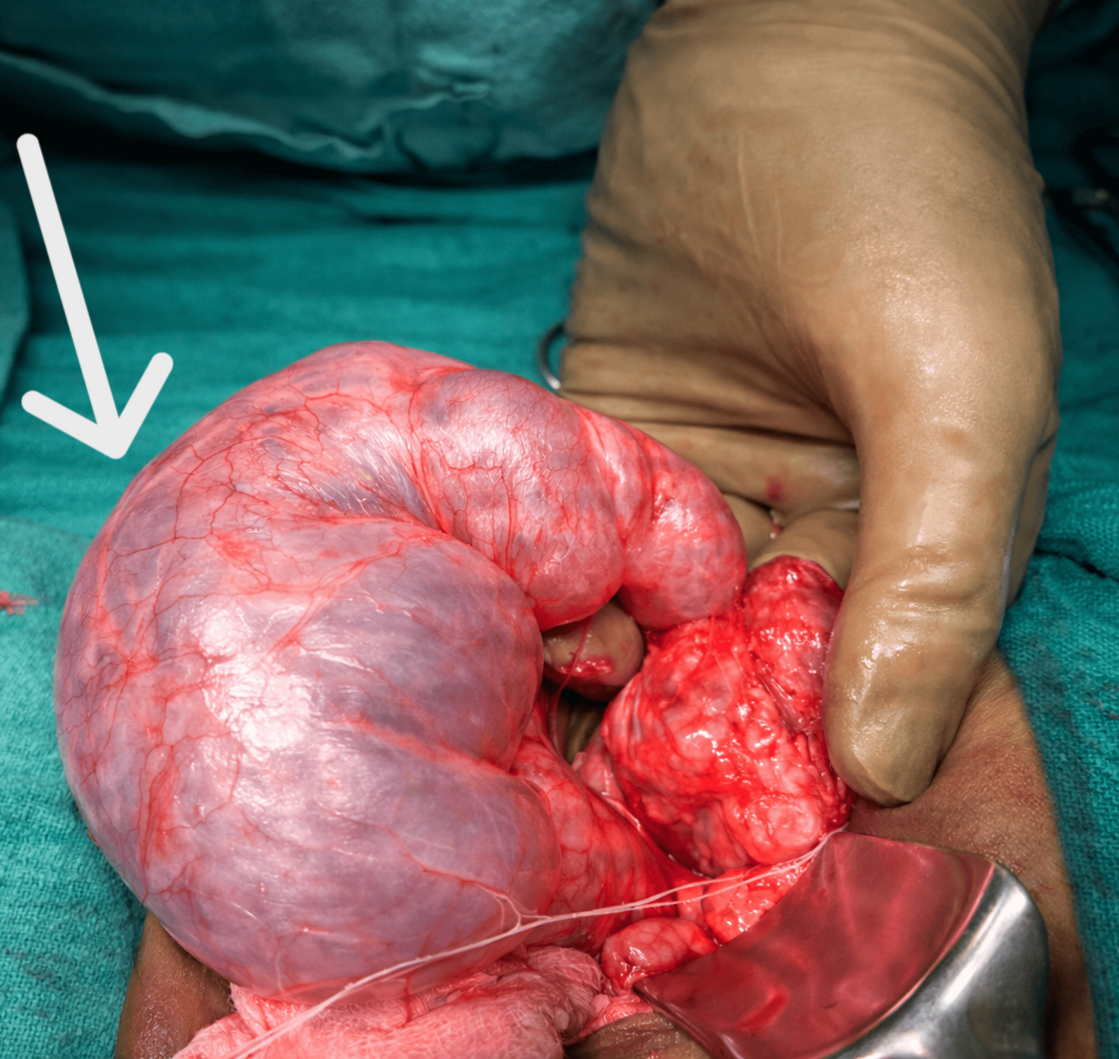
Intraoperative photograph showing segmental dilatation of the mid-ureter (white arrow) Intraoperative image demonstrating a markedly dilated segment of the mid-ureter, with a thinned-out wall and prominent surface vasculature. The dilated segment is seen compressing the adjacent sigmoid colon, correlating with the preoperative findings of large bowel obstruction.

The mass was found to be arising from the left ureter. The proximal and distal ureteric segments were normal in calibre, with visible peristalsis; however, the mid-ureteric segment was markedly dilated (>7 mm) and stenotic, and a guidewire could not be negotiated across either end (Figure 2).

The cystic ectatic mid-ureteric segment was excised and sent for histopathological examination. Uretero-ureterostomy was performed over a 3 Fr, 16 cm double-J stent, using interrupted 5-0 polydioxanone sutures. The abdomen was closed in layers, with subcuticular skin closure.

Postoperative course and histopathology

**Figure 3:**
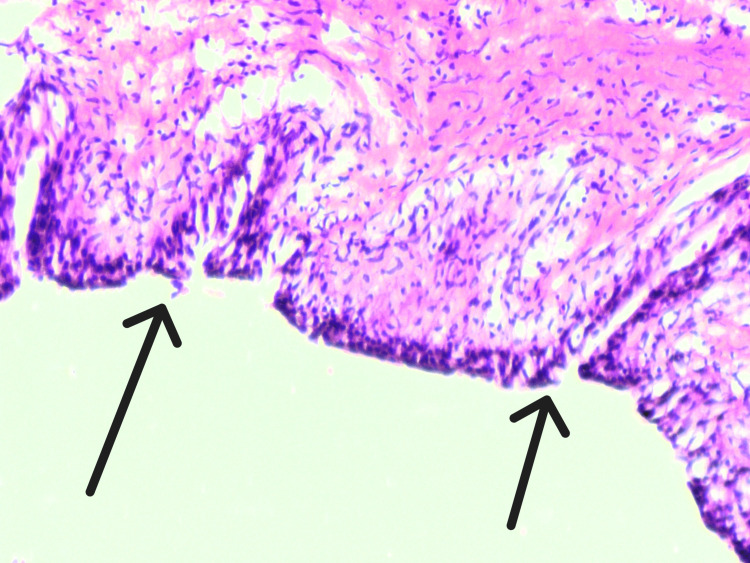
Histopathological features showing muscular hypoplasia with stromal fibrosis Hematoxylin and eosin (H&E)-stained section demonstrating a hypoplastic muscularis layer (black arrows) with associated stromal fibrosis. The muscular layer appears markedly thinned and replaced by fibrotic stroma (original magnification ×200).

The postoperative period was uneventful. Histopathological examination revealed a hypoplastic muscular layer with stromal fibrosis (Figure 3). Follow-up imaging demonstrated complete resolution of hydronephrosis.

## Discussion

Segmental ureteric dilatation is an extremely rare congenital anomaly, with only a limited number of cases reported in the literature [3]. It may be associated with megacalycosis, duplication of the collecting system, and hypoplastic, dysplastic, or non-functioning kidney. The distal ureter may be normal, stenotic, or atretic. Histologically, the dilated segment typically shows a disorganized or dysplastic muscular layer and may be lined by columnar epithelium rather than normal transitional urothelium, findings similar to those observed in the present case.

Mandell et al. described four cases of congenital megacalycosis associated with ipsilateral segmental megaureter in 1987 [1]. In their series, distal ureteric dilatation resembled primary obstructive megaureter, caused by a narrowed, aperistaltic distal ureteral segment, rather than true isolated segmental dilatation. Ramaswamy et al. later reported a congenital segmental megaureter with sparing of the proximal and distal ureteric segments, comparable to our patient [2]. Pinter et al. subsequently documented bilateral involvement in 1997 [6], and Prieto et al. described marked mid-ureteral dilatation with a normal distal ureter [7].

The present case is notable for its clinical presentation mimicking congenital megacolon. The infant presented with abdominal distension and constipation suggestive of Hirschsprung disease; however, intraoperative findings demonstrated extrinsic compression of the sigmoid colon by a stenotic, segmentally dilated mid-ureter. Most previously reported cases present with urinary symptoms or hydronephrosis, rather than intestinal obstruction.

Management depends on ipsilateral renal function and the length of the normal ureteric segment. When adequate proximal and distal ureter are present, excision of the abnormal segment with primary uretero-ureterostomy is the preferred treatment, as performed in our case. Nephroureterectomy is reserved for poorly functioning or dysplastic renal units.

This case emphasizes that infants presenting with intestinal obstruction should also be evaluated for urinary tract anomalies, particularly when imaging demonstrates hydronephrosis.

## Conclusions

Segmental mid-ureteric ectasia with stenosis is a rare congenital anomaly and may, unusually, present as large bowel obstruction. In infants with a restricted abdominopelvic space, extrinsic compression of the colon by a dilated ureter can mimic Hirschsprung disease. Therefore, urinary tract anomalies should be considered in infants presenting with features of intestinal obstruction, particularly when imaging suggests associated hydronephrosis.
